# #Stayathome If You Have a Cold: High SARS-CoV-2 Salivary Viral Loads in Pediatric Patients with Nasopharyngeal Symptoms

**DOI:** 10.3390/v15010081

**Published:** 2022-12-28

**Authors:** Alice Monzani, Cinzia Borgogna, Daniela Ferrante, Benedetta Ciacchini, Enrico Felici, Marisa Gariglio, Ivana Rabbone

**Affiliations:** 1 Division of Paediatrics, Department of Health Sciences, University of Piemonte Orientale, 28100 Novara, Italy; 2 Virology Unit, Department of Translational Medicine, University of Piemonte Orientale, 28100 Novara, Italy; 3 Medical Statistics, Department of Translational Medicine, University of Piemonte Orientale, 28100 Novara, Italy; 4 Pediatric and Pediatric Emergency Unit, The Children Hospital, AO SS. Antonio e Biagio e Cesare Arrigo, 15100 Alessandria, Italy

**Keywords:** viral load, saliva, SARS-CoV-2, COVID-19, rhinitis, pharyngodynia, children

## Abstract

The choice of the best SARS-CoV-2 detection approach is crucial to predict which children with SARS-CoV-2 are at high risk of spreading the virus in order to manage public health measures and policies. In this prospective observational study of 35 children admitted to the Pediatric Emergency Departments of two tertiary hospitals in Northern Italy who tested positive for SARS-CoV-2 by standard RT-PCR in nasopharyngeal swab (NPS), we evaluated their presenting symptoms according to their salivary viral load (SVL) determined by droplet digital PCR (ddPCR). Despite an overall low concordance between SARS-CoV-2 detected by salivary ddPCR and NPS RT-PCR (54.3%), when only patients with nasopharyngeal symptoms were analyzed, the sensitivity of ddPCR in saliva specimens increased to 71.4%, and over half of these patients had high SVL (>10^5^ copies/mL), which was significantly more frequent than in children without nasopharyngeal symptoms (57.1% vs. 14.3%, OR = 8, CI 95% 1.28–50.03, *p* = 0.03). All asymptomatic children had low SVL values. Our findings support the hypothesis that children with nasopharyngeal symptoms are at higher risk of spreading SARS-CoV-2 due to their high SVL and, conversely, asymptomatic children are unlikely to spread the virus due to their low SVL, regardless of their NPS positivity.

## 1. Introduction

Predicting which children with SARS-CoV-2 are at high risk of spreading the virus would be useful for managing public health, reaching a proper balance between COVID-19 containment and the limitation of common life activities, but this relies on choosing the best SARS-CoV-2 detection approach. The current gold standard for SARS-CoV-2 detection is RT-PCR amplification of viral genes from nasopharyngeal swab samples (NPS), although saliva has also been explored as an alternative [1,2]. In pediatric patients, the reported concordance rates between upper respiratory tract swabs and saliva have been highly variable (52.9–89.5%) [1,3,4,5], with better performances in older children and after a shorter duration of symptoms [1,5]. However, the association between COVID-19 symptoms and SARS-CoV-2 salivary viral load (SVL) in children is poorly defined. We therefore examined the clinical features of a cohort of children who tested positive for SARS-CoV-2 by standard RT-PCR in NPS samples according to their SVL determined by droplet digital PCR (ddPCR).

## 2. Materials and Methods

This was a prospective observational study of patients admitted to the Pediatric Emergency Departments (ED) of two tertiary hospitals in Novara and Alessandria, Piedmont, Italy between November 2020 and May 2021, when the Alpha variant was the main circulating SARS-CoV-2 variant in Italy (https://www.epicentro.iss.it/coronavirus/pdf/sars-cov-2-monitoraggio-varianti-rapporti-periodici-19-maggio-2021.pdf, accessed on 24 December 2022). A ddPCR test was performed on saliva specimens from non-vaccinated pediatric patients (0–15 years) with a SARS-CoV2-positive NPS. SVL assessed by ddPCR was correlated with clinical symptoms at admission and the NPS RT-PCR cycle threshold (Ct) values. Parents or guardians provided written informed consent, and the local ethics committee (Comitato Etico Interaziendale Novara, CE 8/21, Novara, Italy) approved the study protocol.

### 2.1. Demographic and Clinical Data

General information including age, gender, and ethnicity were collected for each patient. The presenting symptoms at ED admission were collected and categorized according to fever, dyspnea, cough, diarrhea/abdominal pain, vomiting, asthenia, headache, rhinitis/pharyngodynia, ageusia/anosmia, exanthema, and conjunctivitis. COVID-19 related symptoms were always asked for at the ED admission, even when they were not the reason for the ED referral. When no symptoms were reported, patients who had been admitted for injuries or foreign body ingestion but tested positive on NPS RT-PCR performed at ED admission were also recorded. Prescribed therapy at discharge or need for hospitalization were also documented. Data were recorded by the investigators in an electronic case report form (REDCap v10.3.3, Vanderbilt University, Nashville, TN, USA).

### 2.2. Study Procedures

NPS and saliva samples were collected on the same day, with no clinical interventions occurring between sample collection. NPS were collected by trained healthcare workers in tubes containing 2 mL of 1x Hanks’ balanced salt solution without phenol red (Thermo Fisher Scientific, Waltham, MA, USA). Samples were taken at any hour of the day and were sent the same day or the next morning to the molecular diagnostics laboratory for RT-PCR analyses. As soon as the NPS tested positive, saliva was collected by a pipette from the sublingual region (at least 1 mL of specimen) and then transferred in a 2 mL sterile tube and sent to the Molecular Virology Laboratory (University of Piemonte Orientale, Novara, Italy) for ddPCR analysis.

### 2.3. SARS-CoV-2 RNA Detection and Quantification in Saliva

Commercially available kits were used to detect SARS-CoV-2 virus in COVID-19 patients [Allplex 2019-nCoV Assay (Seegene, Seoul, Republic of Korea): Xpert Xpress CoV-2 plus (GeneXpert, Cepheid, Sunnyvale, CA, USA); Simplexa COVID-19 Direct (DiaSorin Molecular, Cypress, CA, USA); Viasure SARS-CoV-2 BD MAX System (CERTest Biotec, Zaragoza, Spain)]. Nasopharyngeal swabs were processed according to the manufacturers’ instructions. A specimen was considered positive if the gene target had a Ct < 40 [6]. Only the qualitative result of positivity was initially provided by the molecular diagnostics laboratory and the Ct value was recorded in REDCap at a later time.

For ddPCR, total RNA was extracted from 200 μL of saliva using the QIAamp Viral RNA Mini kit (Qiagen, Hilden, Germany) following the manufacturer’s instructions. SARS-CoV-2 genomic RNA was quantified using the QX200 Droplet Digital PCR System (ddPCR, Bio-Rad, Hercules, CA, USA) using the SARS-CoV-2 Droplet Digital PCR Kit (Bio-Rad, Hercules, CA, USA). SARS-CoV-2 quantification was expressed as copy number/mL of saliva.

### 2.4. Statistical Analysis

Quantitative data are presented as medians and interquartile ranges (IQR). Categorical variables are summarized as counts and percentages. Differences in median were evaluated using the Mann–Whitney test. Associations between categorical variables were tested using Pearson’s chi-squared test or Fisher’s exact test, as appropriate. Odds ratios (OR) and 95% confidence intervals (CIs) were calculated. Pearson’s correlation coefficient was applied to measure the relationship between two quantitative variables. A two-sided *p*-value < 0.05 was considered statistically significant. Analyses were performed using STATA software, v17 (Stata-Corp. 2021. Statistical Software: Release 17.0. College Station, TX, USA: Stata Corporation).

## 3. Results

**Figure 1:**
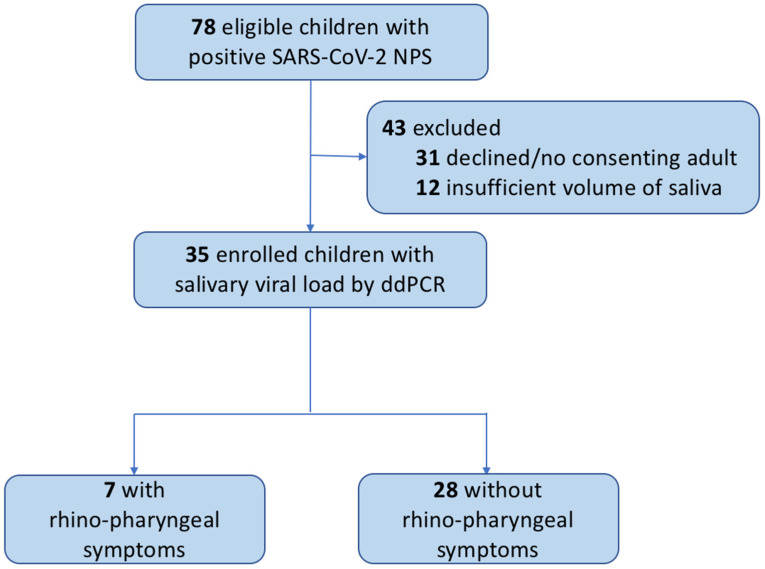
Study flowchart of children and adolescents enrolled in the study.

Of 78 eligible subjects testing positive for SARS-CoV-2 by RT-PCR of NPS samples, 12 were excluded due to insufficient saliva samples and 31 were excluded because consent was not obtained (Figure 1). Thus, SARS-CoV-2 SVL was determined by ddPCR in 35 children (Table 1).

**Figure 2:**
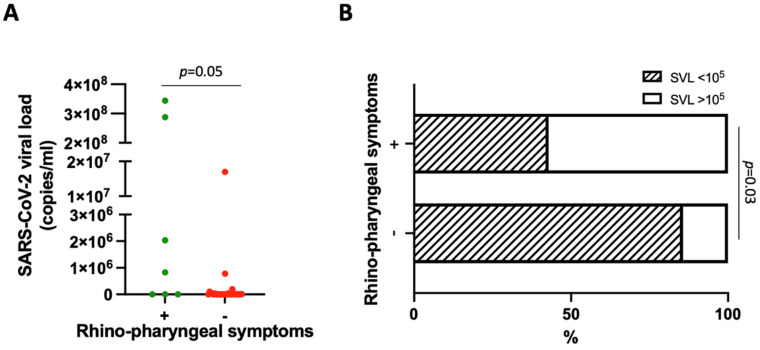
(**A**) SARS-CoV-2 viral load (copies/mL) in patients with [(+): green] and without [(−): red] nasopharyngeal symptoms. (**B**) Percentage of patients with (+) and without (−) nasopharyngeal symptoms displaying SARS-CoV-2 salivary viral load <10^5^ and >10^5^ copies/mL.

Using the NPS results as the reference, the sensitivity of saliva ddPCR was 54.3% (95% CI 37.8–70.8; 19/35 with detectable SVL). In those with nasopharyngeal symptoms (*n* = 7), the sensitivity of salivary ddPCR increased to 71.4% (95% CI 38.0–100.0). The median viral load in saliva by ddPCR was 560 copies/mL (IQR 0–44,400; range 0–344 x 106), which tended to be higher in subjects with rhinitis/pharyngodynia (*p* = 0.05) (Figure 2A). For 30 patients (85.7%), Ct values were available, and the median was 30 (IQR 18–36). SVL values negatively correlated with the Ct values (r = −0.64, *p* = 0.0001) (Supplementary Table S1).

Next, we grouped patients based on their SVL values in subjects with more (“high SVL”) or less (“low SVL”) than 10^5^ SARS-CoV2 copies/mL. This cut-off value was defined according to the mean SARS-CoV-2 viral load found in the saliva of a symptomatic/asymptomatic pediatric cohort [5]. SVL values were high in 57.1% of patients with rhinitis/pharyngodynia, 40% with cough, 33.3% with vomiting, 31.8% with fever, 28.6% with diarrhea/abdominal pain, and 16.7% with dyspnea, but 0% of asymptomatic patients or those with headache, exanthema, or conjunctivitis. High SVL values were significantly overrepresented in subjects with nasopharyngeal symptoms compared with those without these symptoms (4/7, 57.1%, vs. 4/28, 14.3%, OR = 8, CI 95% 1.28–50.03, *p* = 0.03) (Figure 2B).

## 4. Discussion

Here, we report high SARS-CoV-2 SVL in children with rhinitis/pharyngodynia. Despite an overall low concordance between SARS-CoV-2 detected by salivary ddPCR and NPS RT-PCR (54.3%), when only patients with nasopharyngeal symptoms were analyzed, the sensitivity of ddPCR in saliva specimens increased to 71.4%, and over half of these patients had high SVL (>10^5^ copies/mL), which was significantly more frequent than in children without nasopharyngeal symptoms. The overall low concordance between the two techniques is consistent with previous reports, showing high variability between 53% and 100% both in children and in adults [1,3,4,5,7,8]. It could be, at least in part, explained by the lower number of copies per ml in saliva than in swabs and by the variable interval between the onset of symptoms and sample taking [1,4], as shown by Kam KQ et al., who reported that peak SARS-CoV-2 viral loads occurred around day two of illness in infected children [9]. Moreover, four different RT-PCR assays were interchangeably used by our molecular diagnostics laboratory for a diagnostic purpose, and it was not possible to find out which one was used for every test. Indeed, this could represent another possible source of low concordance between saliva specimens and NPS. In our cohort, all asymptomatic children had low SVL values. Taken together, our findings support the hypothesis that children with nasopharyngeal symptoms are at higher risk of spreading SARS-CoV-2 due to their high SVL, and asymptomatic children are unlikely to spread the virus due to their low SVL, regardless their NPS positivity. Our data suggest that salivary ddPCR cannot replace standard NPS RT-PCR for SARS-CoV-2 infection, but it may be useful for stratifying patients with a low or high risk of viral spreading.

In conclusion, children with nasopharyngeal symptoms are likely to spread the virus, as confirmed by their high SVL values and recent data from the delta and omicron variant waves [10,11,12].

## Figures and Tables

**Table 1:** Demographic and clinical characteristics of the enrolled subjects.

		*n* (%)
Gender	Males	17 (48.6)
	Females	18 (51.4)
Age, median (IQR)		8 (2–13)
Ethnicity	White	30 (85.7)
	Black	3 (8.6)
	Hispanic	2 (5.7)
Need for hospitalization	Yes	8 (22.9)
	No	27 (77.1)
Prescribed therapy	None	12 (34.3)
	Antipyretics	20 (57.1)
	Antibiotics	14 (40)
	Steroids	9 (25.7)
Referred symptoms at admission	None	8 (22.9)
	Fever	22 (62.9)
	Dyspnea	6 (17.1)
	Cough	5 (14.3)
	Diarrhea/abdominal pain	7 (20)
	Vomit	6 (17.1)
	Asthenia	1 (2.9)
	Headache	3 (8.6)
	Rhinitis/pharyngodynia	7 (20)
	Ageusia/anosmia	0 (0)
	Exanthema	4 (11.4)
	Conjunctivitis	1 (2.9)

## Data Availability

The data presented in this study are available on request from the corresponding author.

## Supplementary Materials

The following supporting information can be downloaded at: https://www.mdpi.com/article/10.3390/v15010081/s1. Table S1: Clinical data, Ct value from RT-PCR of NPS, and SVL by ddPCR of the enrolled subjects.
