# Supplementary material for: #Stayathome If You Have a Cold: High SARS-CoV-2 Salivary Viral Loads in Pediatric Patients with Nasopharyngeal Symptoms
Source: Viruses. 2022 Dec 28;15(1):81. doi: 10.3390/v15010081 (PMC9867493; doi:10.3390/v15010081)
Supplement: Supplementary file 1 [file viruses-15-00081-s001.zip › viruses-2098408-supplementary.pdf]

Supplementary Table S1. Clinical data, Ct value from RT-PCR of NPS, and SVL by ddPCR of the enrolled subjects.

**Patient #**	**Gender**	**Age**	**Symptoms**	**Days since symptoms onset**	**Ct value from RT-PCR of NPS**	**SVL by ddPCR (copies/ml)**
1	Male	1 month	None	-	33	0
2	Male	1 month	2,8	6	30	0
3	Male	2 years	None	-	42	0
4	Male	13 years	1,4,7	3	36	0
5	Female	8 years	1,10	2	39	0
6	Female	5 years	1,5,10	1	39	0
7	Female	5 years	1,4,10,11	2	37.6	0
8	Female	5 years	1,5	3	33	0
9	Male	6 months	1,4	2	-	0
10	Female	10 years	None	-	-	0
11	Female	6 years	1,3,8	3	13	0
12	Female	7 years	None	-	39	0
13	Female	8 years	1,7	2	30	0
14	Female	6 years	7	1	-	0
15	Male	2 years	1,2,3,4	2	20	0
16	Male	2 years	1,2	2	40	0
17	Female	1 month	1,10	1	21	500
18	Male	16 years	4,5	2	-	560
19	Male	13 years	None	-	28	2730
20	Female	14 years	None	-	31	4450
21	Male	10 years	1,3,8	2	18	4500
22	Female	16 years	None	-	30	6975
23	Male	15 days	1	1	-	17450
24	Female	9 years	None	-	29	25300
25	Male	9 years	1,2	2	30	35500
26	Female	15 years	1,2	2	40	41000
27	Male	14 years	5	1	29	44400
28	Female	10 months	1,4,5	1	13	104800
29	Male	1 month	1,4	2	13	199000
30	Male	13 years	1,2,3	9	30	778000
31	Female	11 months	1,8	1	10	829000
32	Male	11 years	8	1	11	2034000
33	Male	2 years	1,5	2	21	16945000
34	Female	13 years	1,3,8	2	17	287725000
35	Female	15 years	1,8	1	12	344000000

Symptoms: 1. Fever; 2. Dyspnea; 3. Cough; 4. Diarrhoea/abdominal pain; 5. Vomiting; 6. Asthenia; 7. Headache; 8. Rhinitis/pharyngodynia; 9. Ageusia/anosmia; 10. Exanthema; 11. Conjunctivitis
